# Supplementary material for: A Lower CD4 Count Predicts Most Causes of Death except Cardiovascular Deaths. The Austrian HIV Cohort Study
Source: Int J Environ Res Public Health. 2021 Nov 28;18(23):12532. doi: 10.3390/ijerph182312532 (PMC8656512; doi:10.3390/ijerph182312532)
Supplement: Supplementary file 1 [file ijerph-18-12532-s001.zip › ijerph-1404642-supplementary.pdf]

International Journal of
Environmental Research and Public Health

MDPI

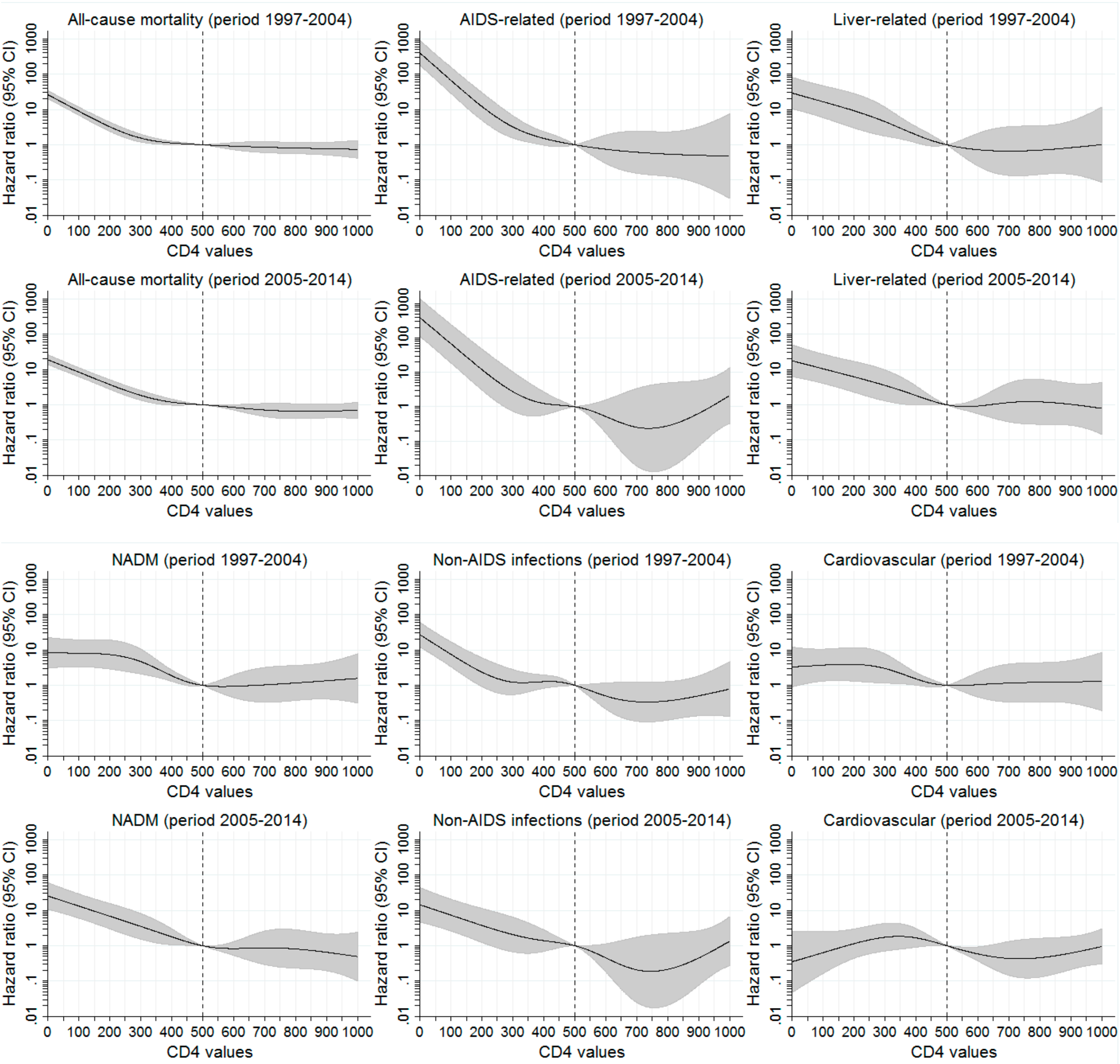

Figure S1. Associations of latest CD4 counts with all-cause mortality and specific causes of death regarding two time periods. Results from adjusted Cox regression models.
